# Differential expression of “*Candidatus* Liberibacter solanacearum” genes and prophage loci in different life stages of potato psyllid

**DOI:** 10.1038/s41598-024-65156-4

**Published:** 2024-07-15

**Authors:** Esmaeil Saberi, Jawwad A. Qureshi, Judith K. Brown

**Affiliations:** 1 School of Plant Sciences, The University of Arizona, Tucson, AZ USA; 2 Department of Entomology and Nematology, IFAS, Southwest Florida Research and Education Center, University of Florida, Immokalee, FL USA

**Keywords:** Insect vector, Psyllid-Liberibacter interactions, Tomato vein-greening disease, Zebra chip disease, Molecular biology, Plant sciences

## Abstract

Psyllid species, including the potato psyllid (PoP) *Bactericera cockerelli* (Sulc) (Triozidae) serve as host and vector of “*Candidatus* Liberibacter spp.” (“*Ca.* Liberibacter”), which also infects diverse plant hosts, including citrus and tomato. Psyllid transmission of *“Ca.* Liberibacter” is circulative and propagative. The time of “*Ca.* Liberibacter” acquisition and therefore vector life stage most competent for bacterial transmission varies by pathosystems. Here, the potato psyllid-“*Ca*. Liberibacter solanacearum” (CLso) pathosystem was investigated to dissect CLso-prophage interactions in the tomato plant and PoP-psyllid host by real-time quantitative reverse transcriptase amplification of CLso genes/loci with predicted involvement in host infection and psyllid-CLso transmission. Genes/loci analyzed were associated with (1) CLso-adhesion, -invasion, -pathogenicity, and -motility, (2) prophage-adhesion and pathogenicity, and (3) CLso-lysogenic cycle. Relative gene expression was quantified by qRT-PCR amplification from total RNA isolated from CLso-infected 1st–2nd and 4th–5th nymphs and teneral adults and CLso-infected tomato plants in which CLso infection is thought to occur without SC1-SC2 replication. Gene/loci expression was host-dependent and varied with the psyllid developmental stage. Loci previously associated with repressor-anti-repressor regulation in the “*Ca* Liberibacter asiaticus”-prophage pathosystem, which maintains the lysogenic cycle in Asian citrus psyllid *Diaphorina citri*, were expressed in CLso-infected psyllids but not in CLso-infected tomato plants.

## Introduction

*“Candidatus”* Liberibacter species are obligate bacterial pathogens that infect both higher plants and certain species of psyllids (Hemiptera: Psylloidea), respectively^1^. Two economically important species in this genus, “*Ca.* L. asiaticus” (CLas) and “*Ca.* L. solanacearum” (CLso), are associated with economically important diseases of cultivated plants^2,3^. The Asian citrus psyllid (ACP) *Diaphorina citri* (Kuwayama) (Liviidae) is the insect vector of CLas, while CLso is transmitted by the potato or tomato psyllid *Bactericera cockerelli* (Sulc) (Triozidae), hereafter referred to as the potato psyllid (PoP)^1^.

Psyllids are hemimetabolous insects and have morphologically distinct adult and nymphal (juvenile) or ‘immature’ stages in which the mature adult stage is preceded by five nymphal stages. Psyllid-mediated transmission of “*Ca*. Liberibacter spp.” utilizes a circulative, propagative mode and once the bacterium is acquired, e.g., associates with the salivary glands and/or oral cavity from which the bacterium is inoculated to plants, or ‘transmitted’ for the life of the vector^4,5^. However, transmission efficiency varies among the specific psyllid vector and developmental stages, depending on the duration of ingestion and/or salivary gland acquisition^6^ and differences in the mechanisms that mediate the pre-acquisition phase of the transmission pathway among the psyllid vectors of CLas and CLso^5^.

As an insect-vectored phytopathogen, “*Ca.* Liberibacter” spp. have evolved a lifestyle in which they utilize both an animal (insect) and plant host for multiplication^1^. Although speculative, “*Ca.* Liberibacter” may have co-evolved first with the psyllid vector, and secondarily with the plant host after repeated psyllid exposure to plants during feeding and reproduction. Recognition of this unusual lifestyle has led to the hypothesis that “*Ca.* Liberibacter” species benefit from having evolved two long-term survival strategies, which rely on some similar as well as pathosystem-specific bacterial effector-host interactions to facilitate infection of hosts classified in entirely different kingdoms.

Transcriptome and proteome analyses have shown that PoP/ACP psyllid host/vector responses vary for different psyllid developmental stages, with respect to the “*Ca.* Liberibacter”-infection cycle and accordingly, to bacterial accumulation, acquisition, and plant-to-plant transmission efficiency^7–9^. Such studies provide compelling evidence for differences in host plant- and psyllid instar-specific gene expression and point to anatomical and physiological differences between psyllid stages that influence their vulnerability to infection and differences in immune responses to pathogen infection^8,9^. Also, life stage-dependent gene expression patterns and transmission competency (efficiency) of “*Ca.* Liberibacter” by infected psyllids appear inter-related and potentially attributable to putative “*Ca.* Liberibacter” and prophage effectors and the corresponding psyllid host interactors that participate in bacterial adhesion, entry, and psyllid gut invasion, as well as bacterial multiplication, exocytosis, hemolymph circulation, systemic infection, and psyllid salivary gland-mediated bacterial acquisition, all requisite to the propagative, circulative transmission mode, characteristic of fastidious Liberibacter species^10,11^.

Genome sequence comparisons for a growing number of “*Ca.* Liberibacter” species^12^ have facilitated the identification of core and pan-genomic features of Liberibacter species and haplotypes or variants, and functional genomic studies^12^ have implicated subsets of “*Ca.* Liberibacter”-encoded genes uniquely expressed in one or both hosts^13,14^. Consistent with an intracellular bacterial lifestyle, “*Ca.* Liberibacter” species apparently lack a canonical type II, III, and IV secretion system and respective associated effectors, as well as genes encoding extracellular degradative enzymes that are commonly associated with free-living or plant-colonizing bacterial pathogens^15^. Instead, “*Ca.* Liberibacter” species access the intracellular compartments of the psyllid host using an intact type I secretion system (T1SS) and encode the hallmark components of Sec machinery implicated in the Sec-dependent secretion pathway, consisting of Sec-dependent effectors that together with T1SS contribute to pathogenicity through bacterial-mediated host modification following translocation of virulence factors^12,14,16^. However, Liberibacter encode flagellar components associated with classical type III secretion systems plus pilus-related genes, which is classified as a type IV secretion system, and auto-transporters that are characteristically known to be associated with other bacterial type V secretion systems. Further, the prophage-like elements are known to encode predicted pathogenicity factors^12,17–19^. Prophage-like sequences are estimated to account for one-fifteenth of the “*Ca.* Liberibacter*”* genome^18^. Prophage composition and location within the bacterial chromosome varies by species or haplotype^20^, and prophage are known to confer virulence-related and phenotypic effects on the psyllid host. In CLas, an excision plasmid and at least two different prophages are chromosomally integrated, in tandem, within the bacterial chromosome, and both can become lytic in plant infections^21^. The Type I (also, SC1) and Type 2 (also, SC2) prophages are functionally implicated in maintaining a lytic or lysogenic life cycle, respectively^18^. The SC1 prophage encodes putative holin and endolysin loci potentially involved in lysogenic to lytic conversion under stress conditions^21^, while SC2 replicates as a high-copy excision plasmid that encodes a peroxidase, feasibly acting as a lysogenic conversion factor^22^. Similarly, the CLso and “*Ca.* Liberibacter africanus” genomes both harbor integrated prophages analogous to SC1/SC2 identified in CLas populations^15^. Further, in the ACP-CLas pathosystem, the SC1/SC1 prophages (A, B) (and several other types in variable abundance), have been detected in libraries constructed from the psyllid host/vector and in *Citrus* and periwinkle plant^23^. However, the extent to which or whether excision plasmids or prophages undergo replication in plants and in the psyllid vectors is not well studied, albeit, a non-canonical prophage-encoded secretory protein is known to be expressed in CLas-infected plants^19^. Thus, a greater understanding of “*Ca*. Liberibacter” virulence mechanisms could aid in the development of a new strategy for HLB disease management^24^. Although global expression patterns have been analyzed in adult psyllids and/or in “*Ca*. Liberibacter”-infected, compared to uninfected hosts^13,14,22,25^, a gap exists in understanding the connections between “*Ca*. Liberibacter” gene expression patterns in the different psyllid development stages and mechanisms involved in pathogenicity, infection, and transmission at nymphal and/or adult stages^9^.

In this study, differences in the relative expression of 33 CLso-bacterial genes/prophage loci with predicted involvement in CLso-infection of the different developmental stages of potato psyllid e.g., 1st–2nd and 3rd–4th immature instars, and teneral adults, were quantified by real-time quantitative PCR (RT-qPCR) amplification. Understanding the basis for differential expression of predicted bacterial genes and prophage loci can inform functional genomic predictions relevant to CLso- and -prophage interplay that may be involved in modulating the psyllid (insect) host to support CLso systemic infection, a requisite to psyllid vector-mediated CLso transmission to plants, compared to expression of the analogous CLso and prophage genes in tomato plants, the latter of the two hosts, which is thought to support the expression of all the CLso genes and prophage loci, respectively.

## Materials and methods

### Potato psyllid colony establishment and maintenance

The CLso-infected potato psyllid (CLso haplotype A), and or CLso-free potato psyllid, were collected in commercial hydroponic tomato greenhouses located in Arizona in 2012. Potato psyllids are endemic to Arizona and elsewhere in the western U.S., and no permit is required to rear them or conduct these studies. The colonies were established by transferring 20–30 adults (females and males) onto 9–10 leaf stage ‘Roma’ tomato *Solanum lycopersicum* Mill. plants, after which they were reared continuously by serial transfer to tomato plants every 4 weeks. Psyllid colonies were maintained in mesh cages (Restcloud Chengdu, China) under LED lights in an insect-free growth chamber or growth room at 24 ± 2 °C, 50% relative humidity, and 14:10 (L:D) photoperiod. The plants were watered twice per week and fertilized monthly with Peter’s 20–20–20 (N–P–K) water-soluble fertilizer (United Industries, St. Louis, MO) at a rate of 1 cc per liter of water (1 teaspoon/gallon). Both CLso-infected and CLso-free potato psyllid cohorts were identified as the ‘Central’ type based on a mtCOI marker^26^ and both colonies were reared in separate insect-free chambers or growth rooms.

Adult psyllids were tested monthly for CLso infection and to confirm the CLso haplotype A using the Lso SSR-1^27^ and OA2/OI2c primers^28^, respectively. The infection rate for PoP-CLso infected colonies was consistently ~ 95–100%. The CLso-free colonies were monitored monthly for CLso presence and were consistently shown to be negative for CLso-infection.

Psyllids used in the differential expression studies were established by transferring CLso-infected adult psyllids to 7–8 leaf stage tomato seedlings and allowed to feed, mate, and oviposit, simultaneously inoculating tomato plants with CLso. Two weeks post insect release, tomato plants were observed periodically for symptom development and assayed for CLso presence by PCR amplification of the CLso-16S rRNA gene, as described^28^. Post-hatch, the immature offspring were maintained on CLso-inoculated tomato plants to establish sub-colonies of CLso-infected psyllids that were synchronized in age. The latter CLso-infected psyllids in the experiments were born and reared on infected tomato plants. After sub-colony establishment, the different developmental stages were collected from CLso-positive tomato plants, in cohorts of 1st/2nd and 4th/5th immature instars, and teneral adults for qRT-PCR amplification. Cohorts of adult and 3rd instar psyllids reared in CLso-free colonies were used as negative controls.

### Selection of genes and loci for real-time quantitative RT-PCR analysis

The hypothesis-driven selection of the genes of interest was based on published research results from laboratory and in silico studies^4,8,9^. Thirty-two CLso/CLas bacterial (13) or prophage (20) genes were selected with predicted functions in “*Ca*. Liberibacter” and prophage pathogenicity, including the potential modulation of PoP host defenses, implicated from previous studies of ACP-CLas and PoP-CLso pathosystems (CLas strain psy62, NC_012985.3) and prophage loci (CLas strain UF506, HQ377374.1; CLso-NZ genome reference (NC_014774.1) (Table 1). Initially, candidate CLas and prophage candidates of interest were identified for the better-annotated CLas-ACP pathosystem with results guiding searches for CLso and prophage candidates. To locate and annotate the CLso orthologs, a homology search was carried out using a protein query for selected gene/loci found in CLas strains psy62 (NC_012985.3) and UF506 (HQ377374.1), and subsequently, the CLso-NZ genome reference (NC_014774.1; CLso-A haplotype) was searched using tblastn and blastx with cut off E-value of ≤ 0.00 and e − 5, respectively. The top 10 BLAST hits for each sequence were considered, and the functional annotations were determined using online bioinformatic tools and/or sequence databases available at the NCBI GenBank website (https://blast.ncbi.nlm.nih.gov/), UniProtKB-Swiss-Prot (https://www.expasy.org/resources/uniprotkb-swiss-prot), the NCBI Conserved Domain search (https://www.ncbi.nlm.nih.gov/Structure/cdd/wrpsb.cgi), conserved protein domain identification by InterProScan (http://www.ebi.ac.uk/InterProScan), and predicted motifs identified by PROSITE Scan (http://npsa-pbil.ibcp.fr/cgi-bin/npsa_automat.pl?page=npsa_prosite.html). The coding sequence having the highest BLAST similarity score and coverage, together with the most biologically relevant annotation, was further verified by an amino acid homology search for each bacterial chromosomal gene or prophage locus, respectively.

**Table 1:** Predicted bacterial chromosomal genes and CLso-associated prophage loci analyzed in this study.

Phage Locus^a^	Predicted function/annotation	CLas homolog^b^	CLso-A homolog^c^	E value^d^
CLso-A-associated phage genes				
AZph1	Phage structural protein	SC1_gp30	DJ66_RS00660	2.00E-18
AZph2	Endolysin protein	SC1_gp035	DJ66_RS05415	7.00E−141
AZph3	Colicin IA; Toxin domain	SC1-gp060	DJ66_RS05400	4.00E−63
AZph4	Colicin IA; TolA domain	SC1-gp060	DJ66_RS05400	4.00E−63
AZph5	Integrase/recombinase	SC2_gp065	DJ66_RS05405	3.00E−07
AZph6	Integrase/recombinase	SC2_gp065	DJ66_RS00645	3.00E−08
AZph7	Major capsid protein	SC1/2_gp090	DJ66_RS00625	0
AZph8	Peroxidase	SC2_gp095	DJ66_RS01460	6.00E−17
AZph9	Glutathione peroxidase	SC2_gp100	DJ66_RS01465	6E−15
AZph10	Phage-related repressor C2	SC2_gp125	DJ66_RS05360	1E−74
AZph11	Phage-related repressor C2	SC2_gp125	DJ66_RS01090	3E−49
AZph12	CRISPR/Cas protein (Cas4)	SC1/2_gp195	DJ66_RS01570	0.00
AZph13	CRISPR/Cas protein (Cas4)	SC1/2_gp195	DJ66_RS05295	0.00
AZph14	Phage anti-repressor	SC1/2_gp200	DJ66_RS05290	1.00E−110
AZph15	Phage anti-repressor	SC1/2_gp200	DJ66_RS00565	8.00E−78
AZph16	Phage anti-repressor	SC1/2_gp200	DJ66_RS01575	8.00E−58
AZph17	DNA polymerase A	SC1/2_gp210	DJ66_RS05280	0.00
AZph18	Colicin immunity protein	SC2_gp255	DJ66_RS06360	4.00E−29
AZph19	Trimeric autotransporter adhesin	SC2_gp240	Un known	3.00E−23
AZph20	Autotransporter	SC2_gp020	DJ66_RS05330	2.00E−08
Bacterial CLso-A chromosomal genes				
AZch1	Phage repressor protein C1	CLIBASIA_01645	DJ66_RS04460	7.00E−113
AZch2	LuxR transcriptional regulator	CLIBASIA_02905	DJ66_RS03455	4.00E−156
AZch3	LysE translocator	CLIBASIA_04415	DJ66_RS01365	3.00E−91
AZch4	Serralysin	CLIBASIA_01345	DJ66_RS04950	0.00
AZch5	Imelysin domain protein	CLIBASIA_02610	DJ66_RS04795	0.00
AZch6	Flagellin; *FliC*	CLIBASIA_02090	DJ66_RS00075	2.00E−152
AZch7	Flagellar hook protein	CLIBASIA_02050	DJ66_RS00115	3.00E−128
AZch8	*Flp3* major fimbrial protein	CLIBASIA_03105	DJ66_RS03635	2.00E−11
AZch9	*TolC*; transporter	CLIBASIA_04145	DJ66_RS02195	0.00
AZch10	Liberibacter unique protein	CLIBASIA_04540	DJ66_RS00995	2.00E−62
AZch11	Tyrosine/serine phosphatase	CLIBASIA_03975	DJ66_RS05455	2.00E−74
AZch12	ABC transporter/ATPase	CLIBASIA_RS04720	DJ66_RS00825	9.00E−145
AZch13	Disulfide bond formation protein	CLIBASIA_RS01775	DJ66_RS04250	1.00E−100
AZch15	*recA*	CLIBASIA_RS00350	DJ66_RS05900	0.00
AZch16	*16S rRNA*	CLIBASIA_RS03560	DJ66_RS00260	0.00
**Locus**	**Predicted function/annotation**	***wDi* homolog**^**f**^	**wBc homolog**^**g**^	**E-value**
Wolbachia endosymbiont of ***Bactericera cockerelli*** gene (loci) ^e^				
AZWo5	Core conserved protein (FtsZ)	HGO53_RS03885	Accession no	0.00
AZWo6	*Wolbachia* repressor protein^h^	WDIAC_RS0101550	Accession no	3.00E−41

^a^Loci analyzed for “*Candidatus* Liberibacter solanacearum” (CLso) haplotype A were designated as “AZph” and “AZch” for predicted bacterial chromosomal genes and prophage loci, respectively.

^b^Selected chromosomal genes corresponding to the *“Ca.* Liberibacter asiaticus” (CLas) reference, UF506 (HQ377374.1) and psy62 (NC_012985.3), respectively.

^c^Predicted homolog of CLso haplotype A sequence (NZ1; NZ_JMTK01000001-5.1) used as the source of chromosomal gene sequences.

^d^The CLso homologs identified from a Blast search of annotated CLas genes against the CLso the LsoNZ genome reference sequence (NC_014774.1) based on the tblastn algorithm. The predicted chromosomal coding region of the potato psyllid ‘Central haplotype’-associated *Wolbachia* endosymbiont, herein ‘AZWo’, from the reference sequence, HGO53_RS03885 and WDIAC_RS0101550.

^f^Predicted homolog of *Wolbachia* endosymbiont of *Diaphorina citri* (strain wDi; CP051608.1).

^g^The genome reference Genbank Accession number is available for the *Wolbachia* endosymbiont of *the potato psyllid*, hence contigs from the laboratory-derived adult PoP transcriptome, which was the source of sequences used in this study (Brown Laboratory, The University of Arizona) was used for the ortholog searches.

^h^A predicted *Wolbachia* repressor-like protein (WP_017531870) with hypothesized involvement in modulating the CLas prophage infection cycle in *D. citri* ^46^.

The predicted CLso orthologs for the CLas-associated prophages SC1 and SC2 (HQ377374.1; Zhang et al., 2011) were identified using viroBlast^29^ and PHAST (PHAge Search Tool)^30^ algorithms with default parameters for homology and prophage region searches, respectively. The orthologs were selected based on the best match among the ten top BLAST hits (viroBlast) having the highest similarity score and coverage with the predicted prophage region of the CLso-NZ genome reference (NC_014774.1), hereafter, “predicted prophage genes” to distinguish them from bacterial CLso chromosomal gene orthologs.

Primers were designed and used to amplify and clone the respective coding regions from total DNA isolated from CLso-A haplotype CLso-infected psyllids, guided by the CDS annotations for CLso-NZ in NCBI (NC_014774.1) genome sequence (Table 1).

### Conventional polymerase chain reaction (PCR) and reverse transcriptase PCR amplification, cloning, and sequencing

Total DNA was isolated from PoP teneral adults (n = 25) using the cetyltrimethylammonium bromide (CTAB) method. Briefly, 600 µl CTAB extraction buffer (2% CTAB; 1.4 M NaCl; 20 mM EDTA; 1 M Tris–HCl pH 8; 0.2% β-mercaptoethanol) was transferred to 1.5 ml safe-lock microtube containing 2.0-mm zirconium beads. Psyllid and plant samples were disrupted using a mini-bead beater (BioSpec Products, Bartlesville, OK, USA) for 2 min and incubated at 65 °C for 20 min. The pellet was collected by centrifugation for 10 min at 12,000 × g. Chloroform (1 vol) was added to the supernatant, followed by centrifugation at 12,000 × g for 10 min at 4 °C. The DNA was precipitated, recovered by centrifugation, washed with 70% ethanol, and resuspended in 60 μl of Milli-Q® water. The DNA quality was determined (O.D. 260/280) and quantified (O.D. 260) using a Nanodrop-2000 spectrophotometer (Thermo Fisher Scientific, Wilmington, DE, United States).

Primers for PCR amplification of CLso-prophage genes from infected psyllids were designed using the IDT Primer Quest Primer Design Tool using the default settings. The primer sequences and annealing temperatures are shown in S1 Table. Reactions (25 μl) contained 2 μl of template DNA, 10 μl 2 × JumpStart REDTaq ReadyMix PCR Reaction Mix (Sigma), and 40 nM of each primer in an Eppendorf Master Cycler Gradient Thermocycler. Cycling parameters were as follows: denaturation for 4 min at 94 °C, 35 cycles for 20 s at 94 °C, 30 s annealing at 55–60 °C (S1 Table), 30-90 s extension at 72 °C, and a final extension of 10 min at 72 °C. Amplicons of the expected size were separated by agarose gel (1%) electrophoresis in 1 × Tris–Acetate-EDTA (TAE) buffer, pH 8.0, and bands were visualized by staining with GelRed (Biotium, USA).

The PCR products were cloned into the pGEM®-T Easy Vector Kit (Promega, Madison, WI), according to the manufacturer’s instructions. Ligation products were transformed into chemically competent *Escherichia coli* DH5α cells. Colonies were assayed by colony PCR amplification to identify those harboring the expected size insert. The plasmid vector containing a cloned fragment of the expected size was isolated from overnight cultures using the GeneJET Plasmid Miniprep Kit (Thermo Scientific). The concentration and purity of DNA plasmid were determined using the nanodrop spectrophotometer (ND-1000, NanoDrop Technologies, Wilmington, DE, USA). The inserts were sequenced bi-directionally (Sanger) (Eton Biosciences, Inc.) with universal primers, M13R and M13F. The sequences were assembled and annotated using the respective sequence database for CLso.

### Isolation of RNA, cDNA synthesis, and reverse transcription

Based on the hypothesis that CLso-prophage gene expression is essential for survival and pathogenicity in both the plant and psyllid host, relative expression of CLso-prophage orthologs in the different PoP life stages and CLso-infected tomato leaves was quantified from total RNA purified from CLso-infected and CLso-free psyllids, or from CLso-infected or CLso-free (qRT-PCR negative control) tomato plants.

Total RNA was purified from groups of 25 teneral adults, 50 4th-5th (25 each) immature instars, and 1st–2nd (100 each) immature instars. Psyllids were transferred to a sterile microcentrifuge tube containing 1000 µl TRIzol® Reagent (Invitrogen, Carlsbad, CA) and one sterile 5 mm steel bead, and pulverized in a bead beater for 5 min. The total RNA was isolated according to the manufacturer’s instructions. The RNA quantity and quality were determined based on the 230/260 and 260/280 ratios, respectively, using a Nano-Drop 2000 Spectrophotometer (Thermo Scientific, Wilmington, DE), with concentrations calculated by the A260/A280 ratio (~ 2.0). Quality and integrity were further evaluated by gel electrophoresis of a 2 μl sample on a 1.2% agarose gel in TAE buffer, pH 8.0, with visualization by GelRed (Biotium, USA) staining.

For tomato plants, the youngest fully expanded leaves (~ 100 mg) were collected from the same tomato plant (12–14 leaf-stage plants, 3-weeks post-inoculation) on which the respective psyllid cohort was reared. Leaves were washed with a stream of distilled water to remove psyllid instars and eggs to avoid possible contamination of RNA / cDNA synthesis. The tomato leaves were transferred to a centrifuge tube (2.0 ml), containing 1 ml TRIzol® Reagent. The total RNA was isolated as described above.

The RNA purified from psyllids or plants was diluted with nuclease-free water to a final concentration of 200 ng/µl, and treated with RNase-Free DNase (Invitrogen, Life Technologies, Carlsbad, USA), according to the manufacturer’s instructions. Reverse transcription was carried out using 2 ug of total RNA per 20 μl reaction with the high-capacity cDNA reverse transcription kit (Applied Biosystems, Carlsbad, CA), according to the manufacturer’s instructions.

## Reference gene selection for qRT-PCR analysis

**Figure 1:**
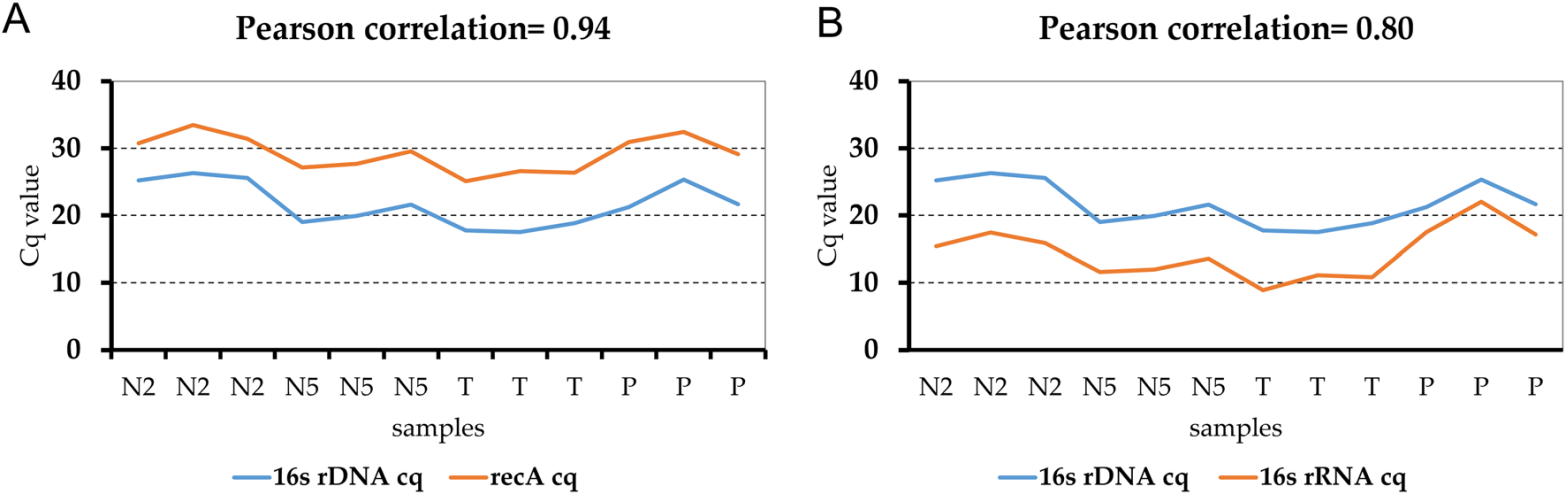
Pearson’s correlation coefficient (r) between “*Candidatus* Liberibacter solanacearum” (CLso) density and expression level of internal reference genes, *recA* (**A**) and *16S rRNA* (**B**), (Cq value) in CLso-infected *Bactericera cockerelli* potato psyllid (PoP) 1st–2nd (N2) and 4th–5th instars (N4), and teneral adults (T4), and tomato plants (P) serving as the CLso inoculum. A robust correlation coefficient (0.9 ≤ r ≥ 1) with CLso genome copy number was observed between the different treatments and expression of *recA*.

Previous analyses have documented the neutral expression of CLso *recA*^31,32^ and 16S rDNA genes^33^, respectively, verifying their utility as reference genes for normalization of gene expression determined by qRT-PCR amplification in the potato psyllid-CLso pathosystem. The relative expression of CLso *recA*^31,32^ and *16S rRNA* were determined by qRT-PCR amplification, and Cq values were compared with CLso genome copy number (Cq), based on Pearson’s correlation coefficient (PCC)^32^. The correlation coefficients for CLso genome copy number and *recA* and *16S rRNA* gene expression were 0.94 and 0.80, respectively, indicating robust correlations (r > 0.95) between CLso genome copy in both immature and adult stages (Fig. 1), with *recA* expression being the most acceptable for all psyllid developmental stages. The *recA* gene was selected as the internal reference gene for normalization of gene expression.

### Quantitative real-time PCR amplification of “*Ca*. Liberibacter” genes

The qRT-PCR reactions were carried out in a CFX96 Touch Real-Time PCR Detection System (Bio-Rad Hercules, CA), according to the manufacturer’s recommendations. For RT-qPCR, three technical replicates were analyzed for each biological replicate (n = 3), in parallel with a no-template (water) control, and a negative control containing no-reverse transcriptase. The primers and probe combinations for CLso and *Wolbachia* spp. mRNA quantification were designed using the IDT Assay Selection Tool available on the IDT website (S2 Table). The PrimeTime® primers and probe were synthesized by Integrated DNA Technologies (IDT). The reaction efficiency for each primer/probe combination was determined by constructing a standard curve for a tenfold series of dilutions ranging from 1 × 10^9^ to 1 × 10^2^ copy number/μl using a cloned fragment of the respective target gene. The plasmid vectors harboring a cloned fragment of the expected size were purified using a Qiagen miniprep kit, linearized by endonuclease digestion (NEBcutter V2.0; http://www.labtools.us/nebcutter-v2-0/), and visualized on a 1% agarose gel. An undigested plasmid containing the respective cloned insert was included as a control to confirm endonuclease activity. The standard curve was derived by plotting the mean cycle quantification (Cq) of the tenfold serial dilutions of linearized plasmid DNA. The slope was used for estimating the reaction efficiency (E) based on the formula: E = 10^ (− 1/slope) − 1.

The RT-PCR amplification reactions (20 μl vol) were carried out in a 96-well plate using amplicon-specific TaqMan probe and primer combinations. Each reaction contained 10 μl of 2X TaqMan Universal Master Mix (Applied Biosystems), 1 μl 20 × Primetime primer/probe stock, 5 μl of RNase-free water, and 4 μl (100 ng/µl; 400 ng) of cDNA template). The qPCR cycling parameters consisted of 50 °C for 2 min initially, 95 °C for 10 min, and 40 cycles of a 2-step program (95 °C for 15 s, 60 °C for 60 s). Three negative control reactions containing no cDNA template were included for each run.

A combination of reference genes for normalization of expression, and a comparison of transcripts expressed in the dual CLso host-system consisting of tomato plants and psyllid vector-insect, the former in which all CLso genes and prophage loci are thought to be expressed, and the latter in which only some of the CLso-prophages are hypothesized to be expressed.

The relative expression of the predicted CLso chromosomal genes and prophage loci were quantified by qRT-PCR amplification from cDNA synthesized from mRNA isolated from immature and adult CLso-infected PoP and from plant CLso-infected tomato plants. The relative fold-change in the expression of the gene of interest was normalized to the *recA* (reference gene) and compared to the tomato sample (calibrator sample), using the formula 2^ΔΔCt^^34^ where ΔΔCt = (C t-target − C t-reference) PoP sample − (C t-target − C t-reference) tomato-sample. Statistical significance (*p* < 0.05) between normalized expression of the gene of interest, relative to the expression of the analogous genes/loci in the tomato plant host was analyzed using the student’s t-test implemented in CFX Maestro 1.1. v. 4.1.2433.1219 software (Bio-Rad). The mean differences between the expression of the gene of interest at different PoP life stages were analyzed by ANOVA, and mean separation was based on Fisher's least significant difference (LSD) test (*p* < 0.05), with InfoStat® software, version 2020.

### Accumulation of CLso in potato psyllid and tomato plants

The number of CLso genome copies was determined by qRT-PCR amplification of CLso *16S rRNA* gene from total DNA isolated from cohorts consisting of 1,2 and 4, 5 nymphal instars, and teneral adult stages, and from tomato plants. Primer and probe design were according to a previously published method for PCR amplification of the CLso 16S rDNA gene^35,36^, present as three copies in the genome^15^. The primers and probe F-CLsoF-qPCR and R-CLso-16S-qPCR-P/HLBr-qPCR (Table S2), were used for real-time quantitative RT-PCR analysis of CLso 16S rRNA in total RNA purified from potato psyllids and tomato plants. The cloned linearized insert was diluted to a final concentration ranging from 10^1^ to 10^10^ in 2 or 40 ng/µl of CLso-free psyllids and uninfected tomato genomic DNA. A standard curve was established for each bacterial gene or phage locus to facilitate quantitative analysis. Copy number was calculated based on the average mass of a DNA base pair of 660 Daltons with the following formula, DNA (copies/ml) = DNA (ng/ml)/(DNA (bp) * 16,109 (ng/g) *660 (Da/bp)/6.02261023 (copies/mole)^35^. The plasmid vector (~ 5* µl* per plasmid with insert) and psyllid and tomato genomic DNA (10 ng psyllid or 200 ng tomato total genomic DNA, respectively) were used as templates for the respective qRT-PCR reaction. Each biological replicate was analyzed in triplicate and the CLso genome copy number was expressed, per 1000 ng of PoP or tomato genomic DNA, respectively.

## Results

### Liberibacter genome copy in tomato plant and psyllid hosts

To explore the molecular mechanism of CLso propagation and circulation in a psyllid host, we measured the CLso bacterial accumulation (genome copy number) and the expression pattern of some related predicted genes associated with CLso propagation and circulation across the different PoP life stages. The CLso genome copy number in CLso-infected tomato plants and from psyllid 1st–2nd instars, 4th–5th instars, and teneral adults was determined by qRT-PCR amplification (Fig. S1). Results indicated an average of 3.0 × 10^5^, 2.7 × 10^3^, 6.4 × 10^5^ and 2.1 × 10^6^ copies/µl in total DNA in tomato plant, 1st–2nd, 4th–5th, teneral adult stages, respectively. Accumulation of CLso was significantly higher in the teneral adults and 4th–5th instars compared to the 1st–2nd instars (F = 17.8, df = 3, *P*-value = 0.0013). There was no significant difference in CLso accumulation (genome copy number) between 4th–5th instars and teneral adults (F = 6.12, df = 1, *P*-value  = 0.06), which is consistent with previously published results^37^ indicating CLso accumulated to lower levels in 1st and 2nd PoP instars, compared to 4th–5th nymphal or adult stage, respectively.

### Expression of CLso orthologs and prophage loci

To investigate if CLso genes are differentially expressed during the different psyllid development stages, the expression of selected CLso-prophage genes/loci was quantified for the different PoP stages, and results were compared to the expression of the analogous genes/loci in the tomato plant host. Thirty-four predicted CLso orthologs that consisted of 13 CLso-A haplotype chromosomal genes, 20 predicted prophage loci, and one predicted *Wolbachia* spp. chromosomal genes were analyzed for relative gene expression for pooled samples of PoP young 1st–2nd, 4th -5th, and teneral adults (Table 1). Five of 34 CLso predicted orthologs analyzed for relative expression exhibited increased expression in the psyllid host, compared to the tomato plant, while 13 of the 34 genes exhibited reduced expression, independent of psyllid life stage, and 15 CLso coding regions were differentially expressed to some extent for all PoP stages examined here (Table 1).

The relative expression of CLso selected genes ranged from ~ 5.97 log2FC (equal to 63- fold-change) for CLso-ortholog of flagellin, *FliC* (AZch6) to 6.6 times lower (equal to -90 times fold-change) for CLso-ortholog of glutathione peroxidase (AZph9) compared to expression of the analogous CLso genes in the tomato plant host.

### Orthologs associated with CLso motility, adhesion, and/or attachment

**Figure 2:**
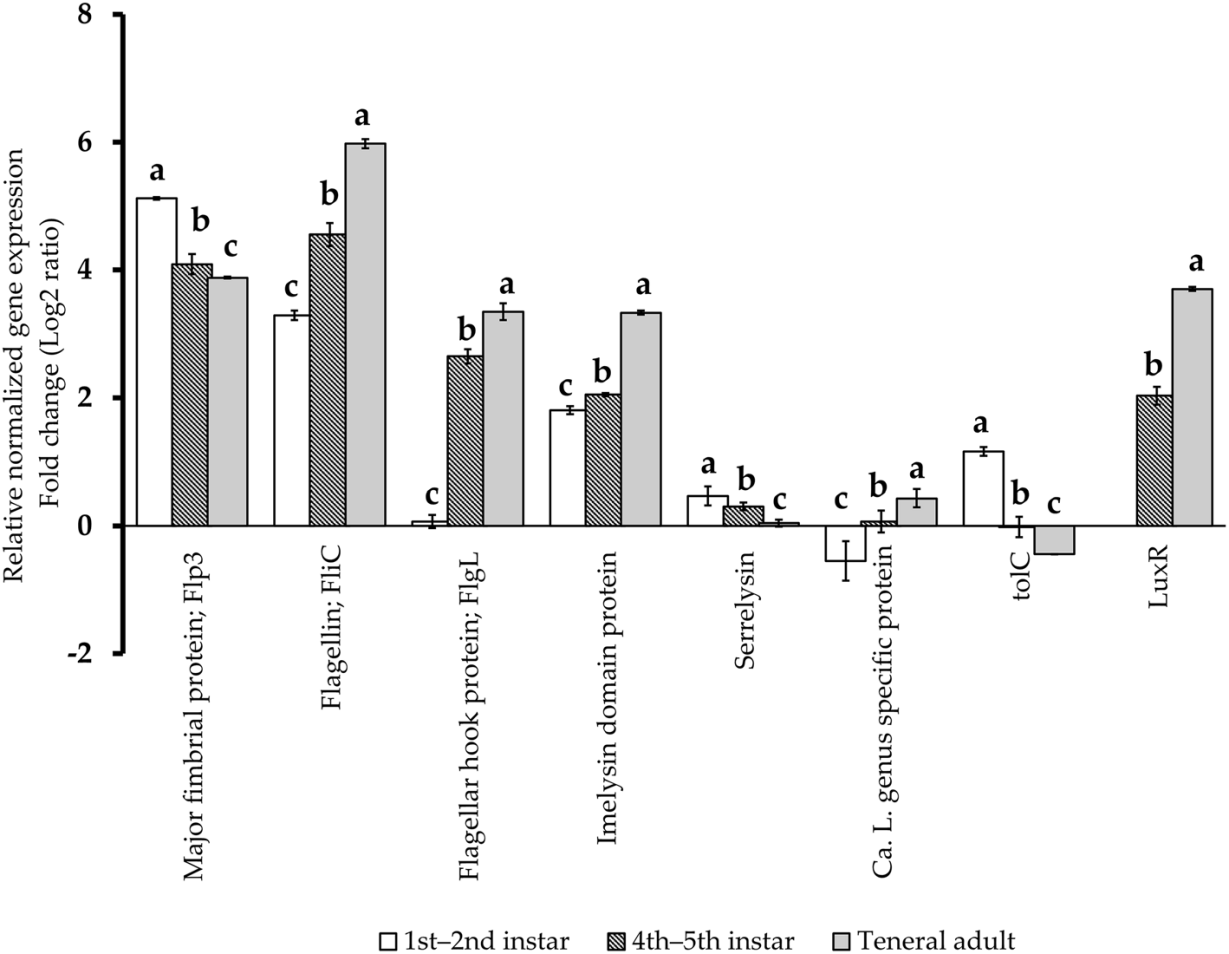
Relative expression of “*Candidatus* Liberibacter solanacearum” (CLso) selected genes in different *Bactericera cockerelli* potato psyllid (PoP) stages, compared to the analogous genes. in CLso-infected tomato plants.. Data were normalized by comparison with the CLso-chromosomal reference gene re*cA.* Error bars represent the standard error of the mean. The different letters indicate a statistically significant difference in expression between the psyllid life stages for which the same gene was analyzed (ANOVA with Fisher's LSD test, *P*-value  < 0.05).

To determine the potential involvement of CLso-predicted pili and flagellar genes in infection of the insect vector and tomato plant host, the expression of pili- and flagellar- genes were analyzed for the different PoP life stages and compared with the expression of the analogous CLso genes in the tomato plant host. The orthologs predicted to encode pili (AZch8) and flagellar (AZch6; AZch7) proteins exhibited increased expression in PoP compared to CLso-expression of the analogous loci detected in CLso-infected tomato plants. Expression of predicted flagellar-associated proteins, *FliC* (F = 377.5, df = 2, *P*-value  < 0.001), and *FlgL* (F = 887.8, df = 2, *P*-value  < 0.001), was high in teneral adults, compared to moderate and low in 4th–5th and 1st–2nd instars, respectively. In contrast, the CLso predicted *Flp3* encoding putative pilus protein (AZch8) was more highly expressed in the first and second instars than in the 4th and 5th instars or teneral adults (F = 161.9, df = 2, *P*-value  < 0.001) (Fig. 2, Table 2).

**Table 2:** Relative expression of selected *“Ca.* Liberibacter solanacearum*”* (CLso) and *Wolbachia* gene (s) in CLso-infected psyllid stages compared to expression of the analogous gene in CLso-infected tomato leaves and CLso-free psyllid stages, respectively.

Locus^1^	Predicted function and annotation	Fold Change ± STDEV^2^		
		1st–2^nd^ instars	4th–5th instars	Teneral adult
AZph1	Phage structural protein	− 0.79 ± 0.07a	− 0.62 ± 0.21a	− 0.95 ± 0.35a
AZph2	Possible Endolysin	− 1.11 ± 0.06a	− 1.02 ± 0.1a	− 1.84 ± 0.36b
AZph3	Colicin IA, Toxin domain	− 0.12 ± 0.35a	− 0.26 ± 0.09a	− 0.93 ± 0.18b
AZph4	Colicin IA, TolA domain	− 2.98 ± 0.24b	− 3.25 ± 0.46b	− 2.54 ± 0.11a
AZph5	Integrase/recombinase	− 1.14 ± 0.25a	− 1.95 ± 0.14b	− 2.55 ± 0.21c
AZph6	Integrase/recombinase	0.31 ± 0.12a	− 0.23 ± 0.15bϕ	− 0.5 ± 0.13b
AZph7	Major capsid protein	− 2.25 ± 0.07a	− 2.46 ± 0.18a	− 4.96 ± 0.15b
AZph8	Peroxidase	− 5.60 ± 0a	− 5.49 ± 0.26a	− 6.09 ± 0.68a
AZph9	Glutathione peroxidase	− 5.22 ± 0.05a	− 6.35 ± 0.1b	− 6.61 ± 0.69b
AZph10	phage-related repressor protein C2	− 0.24 ± 0.1aϕ	− 0.12 ± 0.06aϕ	− 0.17 ± 0.23aϕ
AZph11	phage-related repressor protein C2	0.13 ± 0.03c	0.76 ± 0.03b	0.95 ± 0.15a
AZph12	CRISPR/Cas protein (Cas4)	0.61 ± 0.16a	− 0.43 ± 0.24b	− 1.1 ± 0.24c
AZph13	CRISPR/Cas protein (Cas4)	0.86 ± 0.01a	− 0.55 ± 0.14b	− 1.81 ± 0.16c
AZph14	Predicted phage anti-repressor	− 0.29 ± 0.1bϕ	− 0.94 ± 0.01a	− 0.93 ± 0.02a
AZph15	Predicted phage anti-repressor	− 0.61 ± 0.06a	− 1.44 ± 0.46b	− 2.52 ± 0.11c
AZph16	Predicted phage anti-repressor	− 4.85 ± 0.05b	− 3.71 ± 0.17a	− 5.58 ± 0.33c
AZph17	DNA polymerase A	− 0.56 ± 0.06a	− 1.06 ± 0.23b	− 3.82 ± 0.27c
AZph18	Predicted Colicin immunity protein	− 2.6 ± 0.18a	− 2.75 ± 0.72a	− 3.01 ± 0.18a
AZph19	Autotransporter adhesion gene	1.24 ± 0.14a	− 0.05 ± 0.06bϕ	− 1.79 ± 0.25c
AZph20	Autotransporter; cell wall adhesion	0.39 ± 0.13a	− 1.02 ± 0.19b	− 1.61 ± 0.44c
AZch1	Bacteriophage repressor protein C1	0.44 ± 0.3a	0.35 ± 0.22a	0.49 ± 0.16a
AZch2	LuxR transcriptional regulator	Undetectable CT	2.03 ± 0.14b	3.70 ± 0.03a
AZch3	LysE family translocator	− 1.52 ± 0.12a	− 1.77 ± 0.33a	− 1.77 ± 0.29a
AZch4	Serralysin	0.46 ± 0.14a	0.3 ± 0.06b	0.04 ± 0.05cϕ
AZch5	Imelysin domain protein	1.8 ± 0.06c	2.04 ± 0.02b	3.32 ± 0.02a
AZch6	Flagellin; *FliC*	3.28 ± 0.07c	4.54 ± 0.17b	5.97 ± 0.07a
AZch7	Flagellar hook protein; FlgL	0.06 ± 0.1cϕ	2.64 ± 0.11b	3.34 ± 0.13a
AZch8	Major fimbrial protein; *Flp3*	5.11 ± 0.01a	4.08 ± 0.15b	3.87 ± 0.01c
AZch9	*TolC*; transporter	1.15 ± 0.06a	− 0.02 ± 0.16bϕ	− 0.43 ± 0c
AZch10	Ca. L. genus specific protein	− 0.55 ± 0.21c	0.06 ± 0.17bϕ	0.42 ± 0.13a
AZch11	Serine/tyrosine phosphatase	− 0.12 ± 0.1aϕ	− 0.34 ± 0.1b	− 0.32 ± 0.07ab
AZch12	ABC transporter /ATPase	− 1.43 ± 0.32b	− 0.38 ± 0.11aϕ	− 0.64 ± 0.14a
AZch13	DsbA-like protein	− 0.42 ± 0.05b	− 0.25 ± 0.01aϕ	− 0.54 ± 0.08c
AZWo6	Wolbachia repressor protein	− 0.75 ± 0.02b	− 0. 50 ± 0.09b	0.01 ± 0.00aϕ

^1^Loci analyzed for “*Candidatus* Liberibacter solanacearum” (CLso) haplotype A and were designated “AZph #” and “AZch#” for the prophage and chromosomal genes, respectively. Loci analyzed for the *Wolbachia* spp. endosymbiont of *Bactericera cockerelli* potato psyllid (PoP) ‘Central haplotype’ were designated “AZWo”.

^2^Fold-change (log2 ratio) relative gene expression in CLso-infected psyllid stages, compared to CLso-infected tomato plant and CLso-free psyllid stages, included as references for CLso and the *Wolbachia* endosymbiont of *B. cockerelli*, respectively. Data were normalized to values for the CLso chromosomal reference gene *recA*, and *Wolbachia* spp. *FtsZ*, respectively. The mean values indicated by the same letter are not significantly different (*p* > 0.05). Values representing positive fold-change (log2 ratio) are indicated in bold.

ϕ = No significant difference between normalized expression of the gene of interest, relative to the expression of the analogous gene/locus in the control sample was determined based on the student’s t-test at *p* > 0.05.

### CLso ortholog of prophage genes associated with potential lytic cycle, phage cycle regulation, and lysogenic conversion in immature and adult potato psyllids

**Figure 3:**
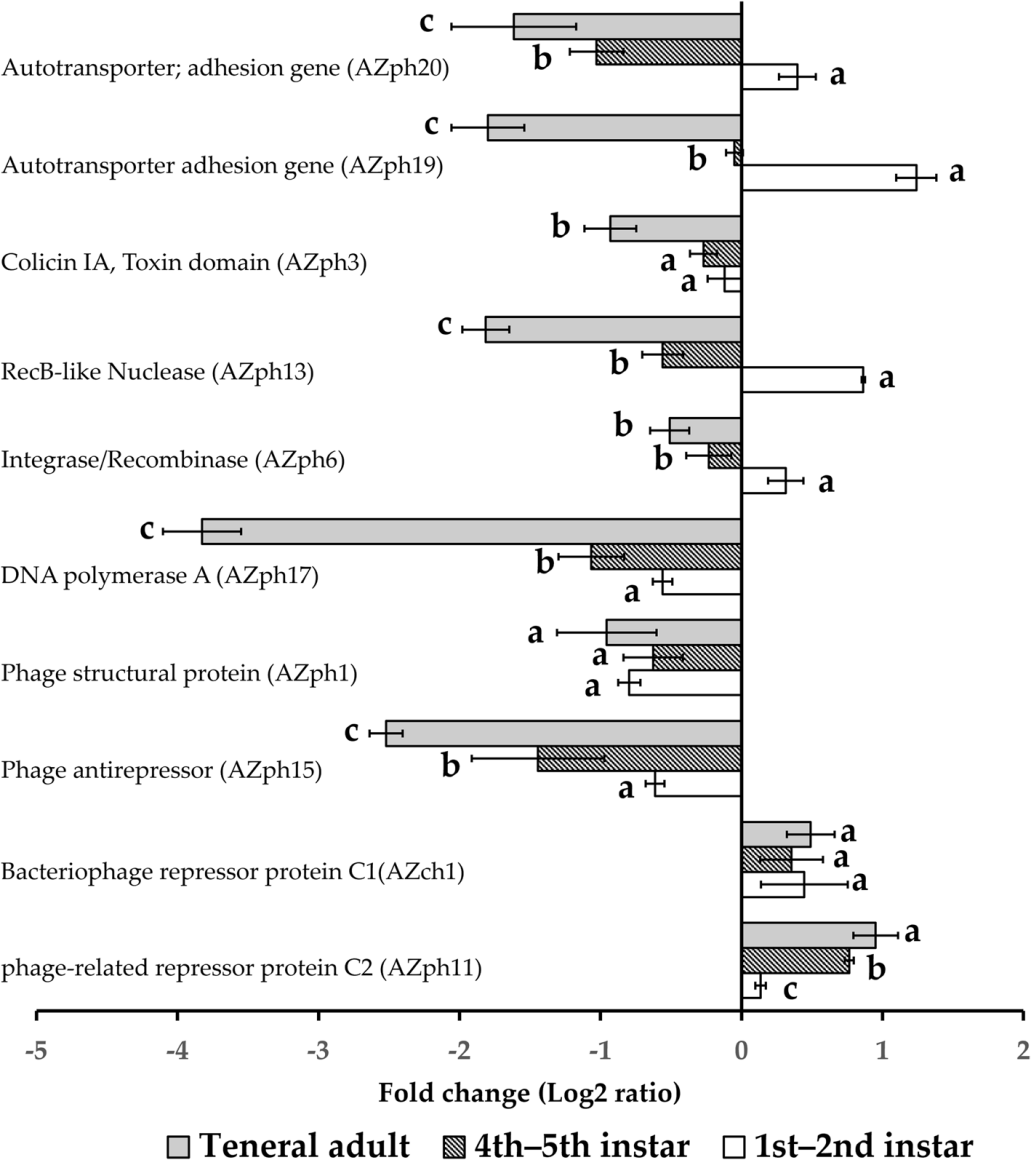
Relative expression of “*Candidatus* Liberibacter solanacearum” (CLso) prophage loci in different potato psyllid life stages. Relative gene expression was calculated using the formula ΔΔCt for real-time quantitative analysis of transcripts detected in the different CLso-infected potato psyllid life stages (insect host), relative to the analogous prophage loci expressed in CLso-infected tomato plants (plant host). Results were normalized using the CLso-chromosomal reference gene re*cA.* The error bar represents the standard error of the mean. Different letters indicate a statistically significant difference in expression between the psyllid life stages for which the same gene was analyzed (ANOVA with Fisher's LSD test, *P*-value  < 0.05).

To determine if the expression of CLso-prophage loci with predicted involvement in lytic and lysogenic infection cycles were indicative of a prophage lytic cycle-induction hypothesis, the expression of selected prophage loci was quantified by qRT-PCR in the psyllid and tomato plant host, respectively. Of particular interest was to determine if the SC1 and SC2 prophages were stably integrated or conversely, activated lysogens, respectively. The CLso prophage orthologs of SC1- and SC2- prophages have been previously predicted to encode lysis, structural, phage cycle regulation (transcription factor), and lysogenic conversion functions (Table 2, Fig. 3). Analysis of prophage genes (orthologs) associated with the lytic cycle, and the predicted structural-(AZph1, AZph7), lysis-(AZph2), and DNA replication-(AZph17) functions, indicated that their gene expression was reduced in all PoP stages, compared to expression *in planta* (Table 2, Fig. 3). Gene expression was significantly different between the teneral adults and nymphal stages for AZph2 (F = 12.4, df = 2, *P*-value  = 0.0074), the loci encoding predicted endolysin and AZph7, predicted major capsid protein (F = 330.5, df = 2, *P*-value  < 0.001), whereas, no differences (F = 1.38, df = 2, *P*-value  = 0.3208) in expression of AZph1 (predicted phage structural protein) were observed for any PoP stages. Finally, significantly decreased expression of the predicted DNA polymerase A, AZph17, was observed in all PoP developmental stages (Table 2, Fig. 3) compared to the expression level observed in tomato plants (F = 208.1, df = 3, *P*-value  < 0.001).

The CLso ortholog locus encoding the predicted prophage anti-repressors, AZph14 (F = 224.6, df = 3, *P*-value  < 0.001), AZph15 (F = 34.5, df = 2, *P*-value  < 0.001), and AZph16 (F = 55.5, df = 3, *P*-value  < 0.001), was expressed at low levels in the nymphal and teneral adults, compared to *in planta* expression. However, the AZph11 locus, encoding the predicted phage-related repressor protein C2, was highly expressed in all psyllid stages, compared to the CLso-infected tomato plant host. Gene expression was higher in the 4th–5th instars and teneral adults, compared to the youngest instars, 1 and 2 (F = 57.19, df = 2, *P*-value  < 0.001) (Table 2). No significant difference (p > 0.05), in expression CLso locus AZph10 encoding the predicted phage-related repressor protein C20 (F = 0.48, df = 3, *P*-value  = 0.6429) was observed for any of the psyllid stages analyzed here, compared to gene expression in the tomato plant host. Interestingly, the analogous CLso chromosomal locus encoding the predicted bacteriophage repressor protein C1 (AZch1) was highly expressed (F = 4.3, df = 3, *P*-value = 0.0459) in all potato psyllid life stages (Fig. 3). The relative expression of the AZph5 phage locus, a predicted integrase/recombinase involved in phage DNA integration, was consistently low for all life stages of PoP (F = 34.27, df = 3, *P*-value  < 0.001) (Table 2). However, nymphal instars 1 and 2 showed greater expression of the integrase/recombinase (AZph6) predicted locus, compared to the tomato plant host (F = 18.37, df = 1, *P*-value  = 0.0011) while 4th–5th instar nymphs and teneral adults showed lower expression level compared to its gene expression in the tomato plant host (F = 13.25, df = 2, *P*-value  = 0.0063) (Table 2) Expression of CLso orthologs encoding RecB-like nuclease, AZph12, and AZph13, which are predicted recombination repair enzymes, and expression of AZph6 were comparable, suggesting they are potential interactors, and so are likely temporarily expressed (Table 2, Fig. 3).

The CLso-A haplotype homologs of CLas-prophage loci AZph8 and AZph9, encoding the predicted lysogenic conversion factor, peroxidase, were expressed in all PoP stages at a significantly lower level than the analogous loci expressed in the CLso-infected tomato plants (AZph8: F = 182.53, df = 3, *P*-value  < 0.001; AZph9: F = 229.14, df = 3, *P*-value  < 0.001) (Table 2). Similarly, the AZph3 and AZph4 loci encoding a predicted toxin-antitoxin system (TA) with predicted colicin IA-like functions, and the predicted cognate colicin immunity protein encoded by the AZph18 locus (F = 40.15, df = 3, *P*-value  < 0.001), showed significantly lower expression in nymphal and teneral adult PoP, compared to expression of the analogous gene in CLso-infected tomato plants (Table 2). Finally, the domain-specific primers-probe combinations provided evidence that the CLso orthologs, TolA (AZph3) (F = 91.62, df = 3, *P*-value  < 0.001), and AZph4, which encode the predicted toxin domain of the colicin IA locus (F = 12.16, df = 3, *P*-value  = 0.002),, were expressed at similarly low levels in all PoP stages examined, compared to expression of the analogous locus in the tomato plant host.

### CLso and prophage gene expression associated with transport of effectors with predicted involvement in virulence and pathogenicity

To investigate selected CLso-prophage genes with predicted functions in “*Ca*. Liberibacter” virulence and pathogenicity, gene expression was quantified for CLso orthologs encoding the predicted metalloprotease serralysin domain, *TolC*, Imelysin, *LuxR*, Liberibacter’ genus-specific secretion protein, and serine/tyrosine phosphatase, in the different PoP life stages and results were compared to expression of the analogous orthologs in the tomato host plant.

The higher expression of CLso ortholog encoding the predicted secreted metalloprotease serralysin domain (AZch4) was observed in the nymphal stages compared to the teneral adults (F = 21.06, df = 2, *P*-value  =  < 0.001). In contrast, relative expression of CLso TolC protein (AZch9) ortholog, the third predicted component of the T1SS I secretion system, was elevated in the 1st–2nd compared to the other PoP stages examined (F = 176.57, df = 2, *P*-value  < 0.001) (Table 2, Fig. 3). Expression of a CLso Imelysin domain protein ortholog (AZch5), a predicted secreted protein^14^ was higher in all PoP stages, compared to the tomato plant host, and relative expression increased significantly, according to the respective PoP instar developmental stage(s) (F = 1050.05, df = 3, *P*-value  =  < 0.001) (Table 2, Fig. 2). Similarly, expression of the CLso ortholog of the locus encoding LuxR (AZch2) was differentially expressed in the mature (4th–5th) instars and teneral adults of PoP, compared to younger 1st–2nd nymph stages (F = 137.44, df = 2, *P*-value  < 0.001) (Table 2, Fig. 2). Expression of the predicted CLso *LuxR* gene was found to increase dramatically in both 4th–5th instars (~ 5 times, fold change) and teneral adults (~ 13-fold change), but not in the 1st–2nd stage of PoP (Fig. 2, Table 2). Expression of the CLso homolog of the predicted ‘Liberibacter’ genus-specific secretion protein (LUSP)^16^, AZch10, increased in teneral adults (F = 29.17, df = 1, *P*-value  = 0.005), compared to the PoP 4th–5th instars, for which no significant differential expression was observed (F = 0.36, df = 1, *P*-value  = 0.58) relative to expression of the analogous genes in the tomato host plant (Table 2, Fig. 2).

The CLso orthologs of predicted serine/tyrosine phosphatase (AZch11), ABC transporter /ATPase (AZch12), LysE family translocator (AZch3), and DsbA-like protein (AZch13) exhibited no differences in expression and/or were expressed at a significantly lower level in PoP, compared to the tomato plant host (Table 2). Differential gene expression of the CLso-prophage homologs of predicted CLas auto-transporters SC2_gp240 (AZph19) (F = 19.58, df = 2 *P*-value  < 0.001) and LasAI (AZph20) (F = 38.49, df = 2, *P*-value  =  < 0.001), was higher in young nymphal instars than for the older nymphal and teneral adults, respectively (Table 2, Fig. 3).

## Discussion

To establish and maintain compatible associations with divergent hosts that require a dual host strategy, Liberibacter spp. must be capable of responding to host-specific signals. Alternative lifestyles and colonization strategies in different host species may therefore be associated with a consequence of such adaptation to two different host environments. In this study, the expression pattern of 33 CLso-prophage genes/loci for the different PoP life stages was characterized and compared with the expression of analogous genes/loci (when expressed) in the CLso-infected tomato plants to investigate if Liberibacter has adopted different strategies to facilitate colonization of the insect (animal) compared to the plant host, and also if interactions differ among the different psyllid life stages when associated with each host, respectively.

Quantification of CLso copy number revealed that bacterial accumulation was significantly higher in the teneral adults and 4th–5th instars, compared to the 1st–2nd instars. No significant difference was observed in CLso accumulation (copy number) between 4th–5th instars and teneral adults. These observations are consistent with previously published results^37^ indicating that CLso genome copy number was lower in the 1st and 2nd instars, compared to the 4th–5th nymphal or adult stages, respectively. Similar levels of gene expression were for the older instars (4th–5th) and teneral adults, with the majority being prophage genes, underscoring important differences especially in prophage activity among the different developmental stages of psyllids (which consist of five nymphal instars, and the adult stage) (Table 1). Previous studies have shown that certain Liberibacter genes are expressed in the psyllid and/or in the plant host, or are predicted to be host-specific^13,14,22,38,39^. Also, differential expression has been reported among the different psyllid stages, and by the plant and psyllid host, respectively^8,9,32^, leading to the hypothesis that different genes may be essential for CLso adaptation and pathogenicity within these different host ‘environments’ i.e. animal *versus* plant^40^.

The expression of predicted CLso gene encoding pili and flagellar assembly proteins revealed that pilin expression in younger psyllid instars was higher than in all other psyllid life stages, while flagellin, and FlgL were more highly expressed in the older immature instars and adult stages, compared to the younger instars. This appears to reflect differences in biological function and/or differences in temporal expression in CLso host developmental stages, which would be consistent with the culmination of the infection cycle in teneral adults to facilitate optimal transmission to the host plant by those teneral adults dispersing to colonize a new host where reproduction will occur. Such patterns of gene expression are consistent with previously recognized functions of bacterial pili and flagella in adhesion and motility, respectively^4,41^. Increased expression of genes associated with the flagellar assembly (flgL, flagellin) and pilin proteins (Flp/Fap) has been reported in the psyllid gut and whole body expression libraries^8,14,32,38^, indicating host attachment utilizing “*Ca* Liberibacter”-encoded surface appendages is integral to psyllid host infection and probably to motility, post-exocytosis from gut cells into the hemolymph and during circulation to salivary glands. Both flagella and a Type IV pilus (T4P) are known to contribute to bacterial pathogenicity in general, and also in fastidious “Ca. Liberibacter” species^15,16^. Previous studies of the CLso-PoP pathosystem have hypothesized a direct involvement of the T4P system in virulence and pathogenesis^24^, specifically, through secretion of “*Ca.* Liberibacter” effector(s) into the host cell. Also, the T4P system is involved in host cell attachment pathosystems^42^ and biofilm formation^43^. In the potato psyllid, dense biofilms have been observed on the gut surface, and filter chamber of 3-5th instars, and adults^4^ with the greatest densities associated with the 4th and 5th instars, and teneral (young) adults^4^. Further, flagella-like surface appendages have been observed in PoP alimentary canals using transmission electron microscopy (TEM)^44^, however, flagellated “*Ca*. Liberibacter” cells have not been reported *in planta*^2^.

Expression of CLso ortholog-prophage loci with a predicted role in lytic cycle induction was lower in PoP stages, compared to that in CLso-infected tomato plants. However, other genes associated with the lysogenic cycle showed increased expression in PoP adults, and in all of the immature PoP psyllid instars analyzed here. These results suggest that lysogeny occurs in the PoP host and that most probably the prophage proteins are also involved in lysogenic conversion and induction of a lytic cycle in the tomato plant host. In this study, and in previously published reports^14,18,32,45,46^, the ”*Ca*. Liberibacter” (ACP-CLas) lytic cycle is activated primarily, if not only, *in planta*. In PoP, lysogenic-lytic cycling of CLas was negligible or undetectable, thereby suggesting the involvement of a predicted repressor-anti-repressor regulatory system, well-known for other bacterial pathosystems to activate the lytic cycle. Although the lysogenic cycle appears to operate in both the tomato plant and PoP host, the lysogenic stage appears to occur overwhelmingly in the tomato plant host, suggesting that ‘leaky” repression may occur toward the end of the infection cycle in the psyllid host^47^. Expression of a small *Wolbachia*-encoded protein was previously hypothesized to regulate the CLas phage lytic cycle in the ACP host^46^ by repressing expression of the *holin* gene in the lytic operon^16^. However, here there was no evidence that a putatively homologous *Wolbachia*-encoded protein was involved in the PoP-CLso infection cycle, as was previously hypothesized for the ACP-CLas pathosystem^46^ in which host-specific expression profiles were implicated in host-switching. To test this hypothesis, the relative gene expression of a predicted homolog of the putative Wolbachia repressor was evaluated for different CLso-infected PoP stage compared to the respective CLso-free PoP samples. The PoP-Wolbachia spp. repressor protein ortholog^46^ was either undetectable or expressed at undetectable low levels in CLso-infected PoP immature and teneral adult stages, respectively, compared to expression in CLso-free PoP (data not showed). This result suggests that the putative *Wolbachia-*encoded repressor is not differentially expressed at a detectable level and/or that the repressor is not involved directly in CLso infection of PoP. Also, the nucleotide and protein blast searches failed to identify CLas *holin* orthologs among CLso genomes available in GenBank, suggesting that ACP and PoP-associated *Wolbachia* spp. may contribute differently to the psyllid (animal) and plant host interactions in this dual-host pathosystem. Notably, the CLso-predicted prophage repressor proteins, AZch1 and SC2_ AZph11, were expressed in adult and immature PoP stages, suggesting that the repressor protein is integral to maintaining the lysogenic cycle in the psyllid host, suggesting there is no need for a *Wolbachia*-like repressor protein in PoP^47^, even though an analogous protein has been implicated in the ACP-CLas pathosystem.

Another predicted repressor, the bacteriophage cI repressor, exhibits variation in tandem repeat number (VNTRs) and frequency among CLas-predicted SC1- and SC2- prophage and CLas-VNTR bacteriophage cI repressor proteins^48^. Further, CLso-prophage-encoded predicted integrase mediates lysogeny through the integration of phage DNA into the bacterial genome^49^. Here, the observed increased expression of CLso orthologs of prophage loci involved in DNA integration (AZph6) and recombination repair (AZph12; AZph13) has provided evidence for prophage DNA integrated in the CLso genome during infection of early immature PoP instar(s), which has been linked to the PoP stages in which the immune system is least compromised^8^. Further, a Cas4 protein is located within the SC1/2_gp195 loci (CLso orthologs of AZph12 and AZph13) (Zheng et al. 2016). This suggests that the Liberibacter CRISPR/Cas system may possibly contribute to superinfection and/or immunity through lysogen expression in the psyllid host.

The higher expression of CLso ortholog of lysogenic conversion genes that encode the peroxidases and TA system implicates their involvement as lysogenic conversion factors that are associated with ‘’*Ca*. Liberibacter’’ pathogenicity in plant host^22,50^, and is consistent with the results of a previous study in which they were predicted to serve as bacterial virulence effectors that suppress host plant immunity^18^. These phage loci may represent examples of genes expressed in both hosts of the ‘’*Ca*. Liberibacter’’-psyllid pathosystem, albeit at different levels and/or possibly, temporally, in the context of the insect and plant infection cycle, respectively.

The results also provided evidence of host-specific expression patterns for several candidate effectors, including Imelysin, for whose expression was also observed in the PoP-CLso pathosystem for all life stages, as well as for the ABC transporter/ATPase, LysE family translocator, and DsbA-like protein, for which expression was higher in tomato plants, compared to the psyllid host, respectively. Also, PoP stage-specific expression was documented for *TolC*, serralysin, LUSP, and *LuxR*, genes with predicted involvement in virulence and pathogenicity. In PoP, *TolC* and serralysin expression levels were similar to those reported for ACP, as well as their higher expression in early immature stages. These observations are consistent with the in silico predictions that serralysin is a TISS substrate^15^. Previous studies have reported that serralysin is expressed in CLas-infected citrus^14^ and in CLso-infected PoP^51^, respectively. Serralysin-like proteins are known to be produced by other plant and human pathogenic bacteria^14^ to aid in circumventing host antimicrobial defenses by inactivating antimicrobial proteins and peptides^52^. Consistent with reduced serralysin expression of predicted immune system-related genes in PoP nymphs (62%) and adults (43%)^8,9^, shifts in defense response genes expression in adult PoP may be attributable to increased expression of developmentally-associated serralysin, a well-characterized virulence factor. Thus, CLso appears to capitalize on the “weak” immune response observed for first through third nymphal instars early in the infection cycle^8,9^.

Consistent with CLso Imelysin transcript levels, CLso accumulation increased concomitantly during maturation of the psyllid host i.e. in the early adulthood stage (Fig. 3, Fig. S1). These results support the possible involvement of a predicted Imelysin-like protein in the uptake of iron from the potato psyllid host by the CLso pathogen, which is consistent with knowledge that Imelysins-belong to a superfamily of bacterial proteins involved in iron uptake^53^.

Up-regulation of LUSP in the PoP teneral stage reveals their probable roles in Liberibacter-psyllid interaction in a time- and/or stage-specific manner. CLso has one copy of these unknown proteins while four homologs of LUSP are predicted in the CLas genome^16^. The LUSP-like proteins may harbor signal peptides (SPs), which prompt cells to translocate proteins to the cell membrane, however, no homolog has been identified by in silico analysis of available microbial databases, suggesting this protein functions as a “*Ca.* Liberibacter”-specific virulence factor^16^.

The plant host-specific expression patterns of CLso orthologs for serine/tyrosine phosphatase, ABC transporter /ATPase, LysE family translocator, and DsbA-like protein were consistent with their involvement in adaptation and survival within the plant but not necessarily in the psyllid (animal) host, respectively. The virulence functions predictions include interfering with the host immune response through protein kinase signaling cascades^16^, uptake and export nutrients e.g., amino acids, lipids, and heavy metal ions^54,55^ and protein secretion^56^.

Members of LuxR family proteins regulate gene expression during quorum sensing to regulate bacterial behaviors, including symbiosis, motility, biofilm formation, and virulence^57,58^, and are involved in insect vector transmission of the bacterium *Xylella fastidiosa*^59^. The coordinated expression of CLso ortholog of predicted *LuxR* and other studied genes associated with motility and virulence observed herein (Fig. 2) suggest that the LuxR transcriptional factor family, may coordinate a density-dependent switch in expression of Liberibacter effectors that provide motility, biofilm formation, and virulence functions.

Here, the developmental stage-specific expression of the CLso-prophage homologs (to CLas), auto-transporters SC2_gp240 (AZph19)^18^ and LasAI (AZph20)^60^, the latter, involved in virulence/transport, was higher in the youngest immature instars (Table 2, Fig. 3). In addition to SDEs, auto-transporters rely on the Sec machinery for transport in “*Ca*. Liberibacter”^18,61^. These loci encode predicted proteins harboring tandem repeats that share homology with proteins identified as leucine-rich repeat-, cell wall-associated biofilm-, and cell surface-proteins^18,61^. The LasAI gene, an auto-transporter^60^, has been implicated in “*Ca*. Liberibacter” acquisition in the psyllid salivary glands^18^. The role of auto-transporters in pathogenic “*Ca.* Liberibacter” species is unknown; however, it seems that they may use an auto-transporter type V secretion system (T5SS) as a substitute secretion system for either a canonical T3SS or T4SS^60^. The increased expression of the predicted auto-transporters, AZph19 and AZph20, in PoP immature instars, suggests that the latter proteins are indispensable for adhesion and colonization that is known to occur early in the CLso-PoP infection cycle, based on this and previous studies of this pathosystem.

## Conclusion

The fastidious nature of “*Ca*. Liberibacter” spp. has limited the understanding of the interactions of Liberibacter spp. The availability of bacterial and psyllid vector genome and transcriptome sequence databases for analyses has aided in the discovery of co-evolved “*Ca.* Liberibacter”-psyllid and -plant host effectors crucial for establishing interkingdom interactions and short and/or long-term survival modes in divergent hosts^8,9,14,31,32^. The results reported here reinforce previous functional genomics-derived hypotheses that the cross-kingdom adaptation of “*Ca.* Liberibacter” will require at least some host-specific bacterial- and prophage-encoded genes, expressed in one host or the other, and/or whose expression is temporally up-or down-regulated in both hosts and in the context of host developmental stage. Prominent among Liberibacter chromosomally-encoded genes were effectors with predicted functions in pathogenicity, including cell wall adhesion-, transporter-, iron-scavenging-, and toxin-anti-toxin- functions, and thiol-disulfide oxidoreductase-like activity (disulfide bond formation motif), previously associated with other bacterial-secreted toxins, virulence factors, adhesion machinery, and motility structures. Also notable were peptidase, phosphatase, and protease activities during early-, mid-, or late processes of CLso-psyllid host invasion, as well as expression of the CLso-prophage autotransporter adhesion protein in the psyllid host, feasibly functioning in gut cell wall adhesion.

This study presents the first in depth analysis of the PoP-CLso-prophage pathosystem that is associated with an herbaceous plant host of CLso. The results underscore similarities and differences with the better-studied ACP-CLas pathosystem, associated with a woody perennial plant host. In both study systems (PoP-CLso, ACP-CLas), “*Ca*. Liberibacter” gene and phage loci expression appear to modulate gene expression of the respective psyllid vector/host, potentially, to some extent through co-coordination of expression of the co-interacting elements of the pathosystem. These results have revealed remarkably fine-tuned, developmental stage-specific gene expression, seemingly orchestrated to facilitate the multiplication of the CLso pathogen to extraordinary levels in the psyllid host, without resultant mortality, or at least, until the bacterial pathogen has been transmitted to its’ plant host.

### Supplementary Information

Supplementary Figure S1.

Supplementary Tables.

## Data Availability

Sequence data have been deposited in the NCBI GenBank database and have been assigned the Accession numbers, as indicated in supplementary Table S1. Primers for amplification of gene/loci sequences are provided. If other experimental details or data not included in the text are of interest to the reader, such information may be requested from the first or corresponding author, respectively.
