# Supplementary material for: Differential expression of “Candidatus Liberibacter solanacearum” genes and prophage loci in different life stages of potato psyllid
Source: Sci Rep. 2024 Jul 15;14:16248. doi: 10.1038/s41598-024-65156-4 (PMC11251058; doi:10.1038/s41598-024-65156-4)
Supplement: Supplementary file 1 — Supplementary Figure S1. [file 41598_2024_65156_MOESM1_ESM.pdf]

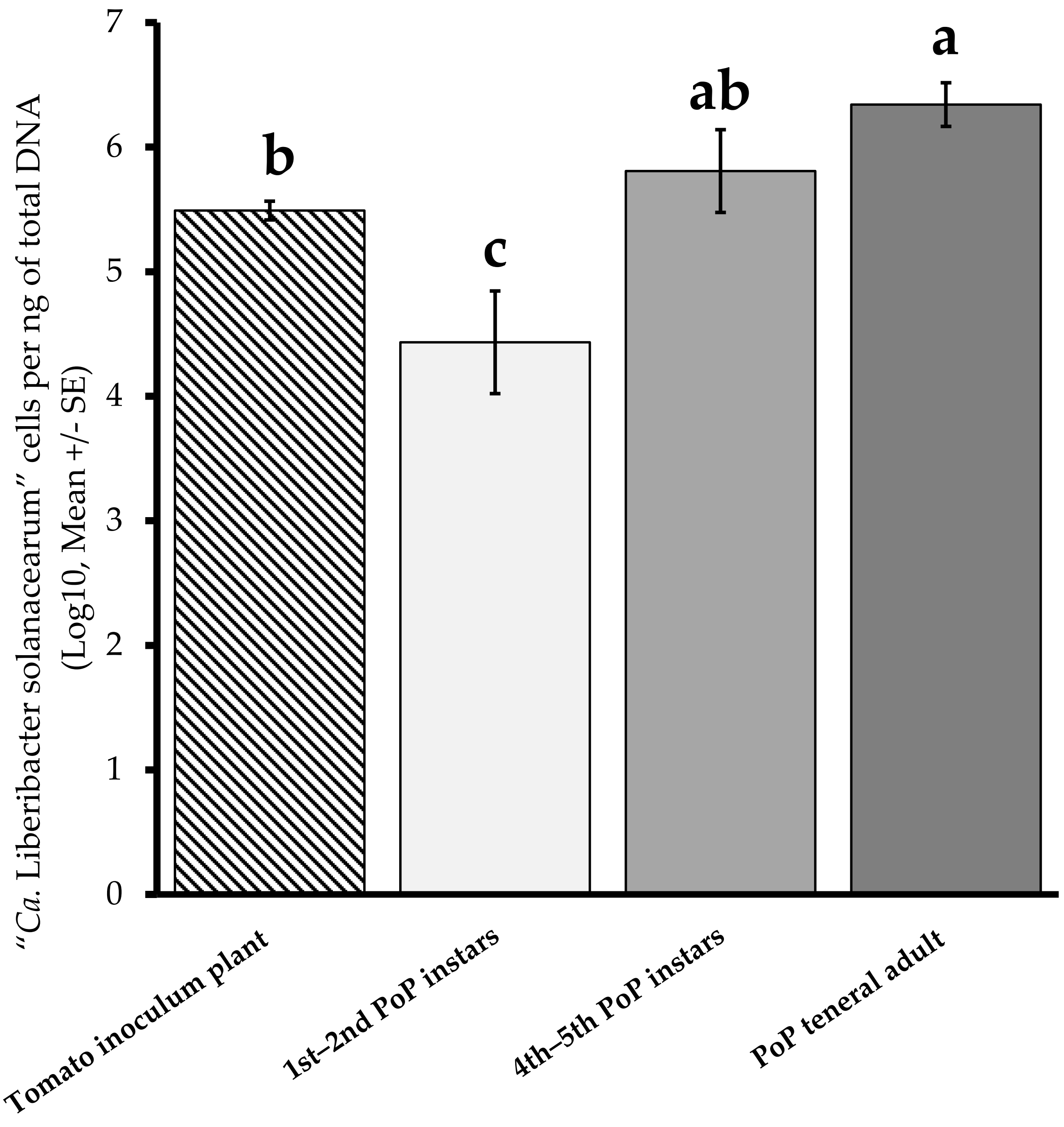

Figure S1. Quantification of “*Candidatus* Liberibacter solanacearum” (CLso) in inoculated tomato plant and CLso-infected *Bactericera cockerelli* potato psyllid (PoP) 1st–2nd and 4th–5th instars, and teneral adults. Error bars represent the standard error of the mean. The means indicated by the same letter(s) for individual samples are not significantly different (ANOVA with Fisher's LSD test, p-value < 0.05).
